# Textbook outcomes in the liver-first approach for colorectal liver metastases: prospective multicentre analysis

**DOI:** 10.1093/bjsopen/zrad123

**Published:** 2024-01-24

**Authors:** José M Ramia, Celia Villodre-Tudela, Laia Falgueras-Verdaguer, Natalia Zambudio-Carroll, José T Castell-Gómez, Silvia Carbonell-Morote, Juan L Blas-Laina, Vicente Borrego-Estella, Belinda Sánchez-Pérez, Mario Serradilla-Martín, RENACI Project Collaborative Study Group, Carlos Domingo-del-Pozo, Gabriel García-Plaza, Francisco J González-Rodríguez, Eva M Montalvá-Orón, Ángel Moya-Herraiz, Sandra Paterna-López, Miguel A Suárez-Muñoz, Maialen Alkorta-Zuloaga, Gerardo Blanco-Fernández, Enrique Dabán-Collado, Miguel A Gómez-Bravo, José I Miota-de-Llamas, Fernando Rotellar, Santiago Sánchez-Cabús, David Pacheco-Sánchez, Juan C Rodríguez-Sanjuán, María A Varona-Bosque, Lucía Carrión-Álvarez, Sofía de la Serna-Esteban, Cristina Dopazo-Taboada, Elena Martín-Pérez, David Martínez-Cecilia, María J Castro-Santiago, Dimitri Dorcaratto, Marta L Gutiérrez-Díaz, José M Asencio-Pascual, Fernando Burdío-Pinilla, Roberto Carracedo-Iglesias, Alfredo Escartín-Arias, Benedetto Ielpo, Gonzalo Rodríguez-Laiz, Andrés Valdivieso-López, Emilio De-Vicente-López, Vicente Alonso-Orduña

**Affiliations:** Department of Surgery, Hospital General Universitario Dr. Balmis, ISABIAL, Universidad Miguel Hernandez, Alicante, Spain; Department of Surgery, Hospital General Universitario Dr. Balmis, ISABIAL, Alicante, Spain; Department of Surgery, Hospital Universitario Dr. Josep Trueta, Girona, Spain; Department of Surgery, Hospital Universitario Virgen de las Nieves, Granada, Spain; Department of Surgery, Hospital Universitario La Paz, Madrid, Spain; Department of Surgery, Hospital General Universitario Dr. Balmis, ISABIAL, Alicante, Spain; Department of Surgery, Hospital Royo Villanova, Zaragoza, Spain; Department of Surgery, Hospital Universitario Lozano Blesa, Zaragoza, Spain; Department of Surgery, Hospital Regional Universitario de Málaga, Málaga, Spain; Instituto de Investigación Sanitaria Aragón, Department of Surgery, Hospital Universitario Miguel Servet, Zaragoza, Spain

## Abstract

**Background:**

Textbook outcome is a valuable tool for assessing surgical outcomes. The aim of this study was to analyse textbook-outcome rates in the prospective Spanish National Registry of the Liver-First Approach (RENACI Project) and the factors influencing textbook-outcome achievement. Additionally, a model for assessing a procedure-specific textbook outcome for the liver-first approach was proposed.

**Methods:**

A retrospective analysis of a prospective and multicentre database that included consecutive patients with colorectal cancers and synchronous liver metastases who underwent a liver-first approach between June 2019 and August 2020 was performed. Two types of textbook outcome were measured: classic textbook outcome and liver-first-approach-specific textbook outcome (which included negative margins, no perioperative transfusion, no postoperative major surgical complications, no prolonged length of hospital stay, no readmissions, no mortality, and full treatment completion). The primary endpoint was textbook-outcome rate for a liver-first approach at 90 days.

**Results:**

A total of 149 patients were included in the analysis. Classic and liver-first-approach-specific textbook-outcome rates were 71.8 per cent (107 patients) and 46 per cent (69 patients) respectively. Factors significantly associated with liver-first-approach-specific textbook-outcome achievement in the multivariable analysis were the number of metastases (OR 0.82 (95 per cent c.i. 0.73 to 0.92); *P* = 0.001) and intraoperative blood loss (OR 0.99 (95 per cent c.i. 0.99 to 1.00); *P* = 0.007). Prolonged length of hospital stay (33 patients, 41 per cent), positive margins (31 patients, 39 per cent), perioperative transfusion (27 patients, 34 per cent), and no full treatment completion (18 patients, 23 per cent) were the items that most frequently prevented liver-first-approach-specific textbook-outcome achievement.

**Conclusion:**

Liver-first-approach-specific textbook outcome is a promising tool for measuring the quality of care when using the liver-first approach for synchronous colorectal liver metastases.

## Teaser

Textbook outcome, an indicator obtained by combining several traditional care variables into a single overall measure, may be considered as a valuable tool for assessing surgical outcomes. The aim of this study was to analyse textbook-outcome rates in the prospective Spanish National Registry of the Liver-First Approach (RENACI Project) and the factors influencing textbook-outcome achievement. Additionally, a model for assessing a procedure-specific textbook outcome for the liver-first approach was proposed.

## Introduction

There are three different strategies for managing patients with colorectal cancer (CRC) and synchronous liver metastases (SCRLM): primary resection followed by liver resection; simultaneous liver and colon resection; and liver resection followed by primary resection^1–3^. This last option, called the ‘reverse strategy’ or the ‘liver-first approach’ (LFA), was proposed in 2006 by Mentha *et al*.^1^ for the management of patients with asymptomatic colorectal tumours with initially unresectable or borderline-resectable liver metastases. The LFA entails performing high-impact chemotherapy first, resection of liver metastases second, and, finally, removal of the primary tumour^1–4^. The complete treatment includes two major surgeries plus chemotherapy/radiotherapy and is associated with high morbidity and mortality^1–4^. A recent paper by the LiverMetSurvey Group for patients with bilobar SCRLM proposed the LFA as the standard, because it achieves excellent short-term results and long survival^2^. No quality metrics have been performed in previous series of the LFA.

Auditing and improving the quality of care are essential components of surgical activity^5–9^. The most commonly used methods for auditing have been assessment of postoperative complications (morbidity and mortality), length of hospital stay (LOS), readmission rate^5,8,10–12^, and failure to rescue, as well as benchmarking^13,14^.

In 2013, Kolfschoten *et al*.^15^ introduced a new tool to assess surgical results called ‘textbook outcome’ (TO), an indicator obtained by combining several variables into a single overall measure (no serious postoperative complications, LOS less than 75th percentile, no mortality, and no readmissions). To obtain a TO, patients must present all the items mentioned^7,8,15–18^.

TO has multiple strengths: the included items are recorded routinely; it is reproducible; and it is clinically meaningful^9^. The lack of procedure-specific items is the main disadvantage of this tool. Moreover, as it is an ‘all-or-nothing’ indicator, it might underestimate surgical morbidity and mortality after complex surgery, such as hepato-pancreato-biliary (HPB) or oesophageal surgery^8,9,18^. To overcome these limitations, procedure-specific TOs have been proposed for pancreas and liver surgery^9,10,12^, but the lack of agreement on the items to be included makes it difficult to compare surgical series^19^. Moreover, most studies combine liver and pancreas surgery or are procedure-specific and/or even disease-specific^3,10,13,16,17^.

The aim of this study was to analyse TO rates in the prospective Spanish National Registry of the Liver-First Approach (RENACI Project) and the factors influencing TO achievement and to propose an LFA-specific TO model.

## Methods

### Design

A retrospective analysis of a prospectively collected data set that included consecutive patients with CRC and SCRLM (defined as the presence of liver metastases at the time of CRC diagnosis) recruited among Spanish hospitals with medium to high HPB surgery volumes from 1 June 2019 to 30 August 2020 was performed. The study coordinators contacted the HPB surgery units of all Spanish hospitals that perform liver surgery. A total of 40 second- and third-level hospitals agreed to participate in the study.

The study protocol was approved by the Ethics Committee of Aragon (Clinical Trials registry: NCT04683783, available at https://clinicaltrials.gov/ct2/show/NCT04683783). All patients were fully informed of the details of the study and provided written informed consent. The ethical principles outlined in the Declaration of Helsinki and Good Clinical Practice were followed.

### Patients

Patients undergoing an elective LFA for SCRLM, greater than or equal to 18 years old, and having an ASA grade of I–III^20^ were included. Patients undergoing an associating liver partition and portal vein ligation for staged hepatectomy (‘ALPPS’) procedure were excluded.

### Treatment strategy

The LFA begins with neoadjuvant chemotherapy for systemic control of the disease. For patients with a partial response or stabilization of liver disease according to Response Evaluation Criteria in Solid Tumours (‘RECIST’)^21^, liver surgery was performed to prioritize the removal of SCRLM. Patients with locally advanced rectal tumours underwent radiotherapy or chemotherapy/radiotherapy, followed by resection of the primary tumour.

### Outcomes

The primary endpoint was the TO rate for the LFA at 90 days. Two types of TO were measured: classic TO (which includes the absence of severe postoperative complications (Clavien–Dindo grade greater than or equal to III)^22^, LOS less than 75th percentile, no mortality, and no readmissions measured at 90 days); and LFA-specific TO (which includes seven liver-surgery-specific items, as defined by Merath *et al*.^8^, measured at 90 days: negative margins, no perioperative transfusion, no postoperative major surgical complications (Clavien–Dindo grade less than III)^22^, no prolonged LOS, no readmissions, no mortality, and full treatment completion (chemotherapy, liver surgery, chemotherapy/radiotherapy, and colon surgery)).

### Study variables

The following variables were studied: age, sex, BMI, ASA grade, and past medical history; clinical symptoms; carcinoembryonic antigen (CEA) and carbohydrate antigen (CA) 19.9 preoperative levels; location of the primary tumour, number, size, and location of liver metastases, and extrahepatic disease; need for stent placement or colostomy, neoadjuvant chemotherapy, and time from diagnosis to start of chemotherapy; portal embolization, two-stage hepatectomy, type of surgery, major (greater than or equal to three segments) and minor (less than three segments) hepatectomy, operating time, approach, intraoperative blood loss, clamping time, R status, degree of tumour regression, postoperative morbidity and mortality (according to the Clavien–Dindo classification)^22^, bile leak, post-hepatectomy insufficiency and haemorrhage defined by International Study Group of Liver Surgery classification^23–25^, LOS, readmissions, adjuvant chemotherapy, and radiotherapy; number of patients with resection of the primary tumour, type of surgery, approach, operating time, intraoperative blood loss, postoperative morbidity and mortality after primary resection, LOS, readmissions, histological type, TNM classification, degree of tumour regression, and adjuvant chemotherapy; and postoperative follow-up (months), death, and recurrence.

### Statistical analysis

Quantitative data are expressed as median (interquartile range (i.q.r.)) or mean(s.d.) and qualitative data are expressed as frequencies and percentages. Differences between groups were analysed using the non-parametric Mann–Whitney *U* test and Pearson’s chi-squared test as appropriate. Fisher’s exact test was used when multiple variables were included. Logistic regression analysis was used to estimate and test factors for their association with TO achievement. A backward strategy was adopted, with a statistically significant cut-off for variable screening of 0.050. Factors associated with TO achievement in the univariable analysis at *P* ≤ 0.050 were included in the multivariable analysis.

Calculations were performed using the programs Microsoft^®^ Excel for Mac, version 16.49 and SPSS^®^ for Mac, version 26.0 (IBM, Armonk, NY, USA). A *P* value <0.050 was considered statistically significant.

## Results

### Overall data

A total of 149 patients underwent surgery using an LFA. The median age was 61 (i.q.r. 52–68) years and 96 (67 per cent) were male (*Table 1*); 76 (51 per cent) patients were ASA grade I and 64 (43 per cent) patients were ASA grade II. The most prevalent location of the primary tumour was the rectum (72 patients, 48 per cent), followed by the sigmoid colon (51 patients, 34 per cent). Of the 72 patients with rectal tumours, 6 (8 per cent) were T1, 13 (18 per cent) were T2, 43 (60 per cent) were T3, and 10 (13 per cent) were T4. In 130 (87 per cent) patients, no therapeutic action was taken before surgery; a diverting colostomy was performed for 9 (6 per cent) patients. A median of three metastases was recorded and the median size of the largest metastasis was 30 (i.q.r. 19–59) mm. In 71 (48 per cent) patients, greater than or equal to four segments were involved. The median time between the diagnosis and the start of treatment was 28 (i.q.r. 17–45) days. A median of 6 (i.q.r. 4–8) cycles of chemotherapy were administered before surgery. There were 60 (40 per cent) and 89 (60) patients who underwent major and minor hepatectomy respectively. The approach used was laparoscopic for 53 (36 per cent) patients and open for 96 (64 per cent) patients. The median LOS after liver surgery was 6 (i.q.r. 4–9) days.

**Table 1:** Characteristics of patients achieving classic and liver-first approach-specific textbook outcomes

Variable	Total	Classic textbook outcome			Liver-first approach-specific textbook outcome		
		Failed	Achieved	*P*	Failed	Achieved	*P*
Number of patients	149	42 (28)	107 (72)	–	80 (54)	69 (46)	–
Age (years), median (i.q.r.)	61 (52–68)	60 (48–70)	61 (55–68)	0.577	64 (52–70)	60 (52–67)	0.078
**Sex**							
Male	96 (64)	28 (67)	68 (64)	0.437	28 (67)	68 (64)	0.437
Female	53 (36)	14 (33)	39 (36)		14 (33)	39 (36)	
Weight (kg), median (i.q.r.)	73 (64–82)	68.5 (63–80)	75 (65–82)	0.226	70 (63–80)	76 (67–85)	0.044
Height (cm), median (i.q.r.)	166 (160–173)	165 (160–173)	168 (162–173)	0.598	166 (160–173)	167 (162–173)	0.829
**ASA grade**							
I	76 (51)	2 (5)	7 (7)	0.427	6 (8)	3 (4)	0.668
II	64 (43)	25 (60)	51 (48)		39 (49)	37 (54)	
III	9 (5)	15 (36)	49 (46)		35 (44)	29 (42)	
**Tumour location**							
Ascending–transverse colon	10 (7)	5 (12)	5 (5)	0.157	7 (9)	3 (4)	0.413
Descending colon	16 (11)	6 (14)	10 (9)		9 (11)	7 (10)	
Sigmoid colon	51 (34)	16 (38)	35 (33)		30 (38)	21 (30)	
Rectum	72 (48)	15 (36)	57 (53)		34 (43)	38 (55)	
**Therapeutic action before surgery**							
None	130 (87)	37 (88)	93 (87)	0.914	68 (85)	62 (90)	0.203
Diverting colostomy	9 (6)	2 (5)	7 (7)		4 (5)	5 (7)	
Others	10 (7)	3 (7)	7 (7)		8 (10)	2 (3)	
Number of metastases, median (i.q.r.)	3 (2–6)	5 (3–8)	3 (1–6)	0.002	5 (2–7)	2 (1–5)	<0.001
Size of the largest metastasis (mm), median (i.q.r.)	30 (19–59)	34.5 (20–65)	27 (16.4–56)	0.218	36 (21–65)	23.5 (13–40)	0.001
**Number of affected segments**							
1	28 (19)	4 (10)	24 (23)	0.013	8 (10)	20 (29)	0.001
2	31 (21)	5 (12)	26 (25)		12 (15)	19 (28)	
3	18 (12)	4 (10)	14 (13)		11 (14)	7 (10)	
≥4	71 (48)	29 (69)	42 (40)		49 (61)	22 (32)	
Time since treatment onset (days), median (i.q.r.)	28 (17–45)	22 (15–42)	29 (19–45)	0.429	28 (18–43)	28 (17–47)	0.893
Number of chemotherapy cycles	6 (4–8)	8 (4–10)	5 (4–7)	0.035	7 (4–10)	5 (4–7)	0.007
**Procedure**							
Right hepatectomy	41 (28)	22 (52)	19 (18)	0.001	27 (34)	14 (20)	0.001
Left hepatectomy	14 (9)	3 (7)	11 (10)		9 (11)	5 (7)	
Segmentectomy	52 (35)	10 (24)	42 (39)		24 (30)	28 (41)	
Right trisectionectomy	3 (2)	2 (5)	1 (1)		3 (4)	0	
Left trisectionectomy	2 (1)	1 (2)	1 (1)		2 (3)	0	
Wedge	36 (24)	4 (10)	32 (30)		14 (18)	22 (32)	
**Major/minor hepatectomy**							
Major hepatectomy	60 (40)	28 (67)	32 (30)	<0.001	41 (51)	19 (28)	0.003
Minor hepatectomy	89 (60)	14 (33)	75 (70)		39 (49)	50 (73)	
**Approach**							
Open	96 (64)	34 (35)	62 (65)	0.006	57 (59)	39 (41)	0.044
Laparoscopic	53 (36)	8 (15)	45 (85)		23 (43)	30 (57)	
Portal embolization	19 (13)	14 (33)	5 (5)	<0.001	15 (19)	4 (6)	0.018
Previous volumetry	19 (13)	14 (33)	5 (5)	<0.001	15 (19)	4 (6)	0.018
Two-stage surgery	19 (13)	10 (24)	9 (8)	0.002	14 (18)	5 (7)	0.065
Length of intervention (min), median (i.q.r.)	240 (186–312)	240 (220–330)	240 (180–300)	0.066	270 (220–335)	210 (180–290)	<0.001
Intraoperative blood loss (ml), median (i.q.r.)	200 (100–400)	360 (150–700)	200 (100–350)	0.018	350 (150–500)	200 (100–300)	0.001
Pedicle clamping time (min), median (i.q.r.)	30 (12–56.5)	35 (10–65)	30 (14–50)	0.488	40 (16–60)	28 (9–45)	0.028

Values are *n* (%) unless otherwise stated. i.q.r., interquartile range.

A total of 17 (11 per cent) patients presented major complications (Clavien–Dindo grade greater than or equal to III), the 90-day mortality was 0.7 per cent, and 15 (10 per cent) patients had type A liver failure, 13 (9 per cent) patients had a postoperative bile leak, 9 (6 per cent) patients had intra-abdominal collections, and 5 (3 per cent) patients required an additional re-intervention (1 patient required percutaneous drainage and the remaining 4 patients required surgery). The classic TO rate was 71.8 per cent (107 patients); after adding the three LFA-specific items, the rate decreased to 46 per cent (69 patients).

### Classic textbook outcome

The patients who achieved a classic TO (107 patients, 71.8 per cent) were compared with those who did not (42 patients, 28 per cent). No significant differences in classic TO achievement were observed regarding patient age or sex. Significant differences were found regarding the number of metastases (3 *versus* 5 respectively; *P* = 0.002), the number of affected segments (for example if greater than or equal to four affected segments, 42 (40 per cent) *versus* 29 (69 per cent) respectively; *P* = 0.013), the number of chemotherapy cycles (5 *versus* 8 respectively; *P* = 0.035), and the type of procedure with TO being more frequent for patients undergoing minor hepatectomies (75 *versus* 14; *P* < 0.001). A total of 45 (85 per cent) patients treated laparoscopically achieved a classic TO (*P* = 0.006), whereas two-stage surgery had lower classic TO rates (8.4 per cent for patients who achieved a classic TO compared with 23.8 per cent for patients who did not achieve a classic TO; *P* = 0.002). Intraoperative blood loss was higher for patients who did not achieve a classic TO (*P* = 0.018).

Univariable analysis showed that number of metastases, number of affected segments, type of procedure, surgical approach, previous portal embolization, two-stage surgery, and intraoperative blood loss could act as significant predictors of classic TO achievement (*Table 2*). In the multivariable analysis, only previous portal embolization reached statistical significance, negatively influencing classic TO achievement (OR 0.23 (95 per cent c.i. 0.06 to 0.93); *P* = 0.042).

**Table 2:** Logistic regression analysis of characteristics associated with a classic textbook outcome

Characteristic	Univariable analysis		Multivariable analysis	
	OR (95% c.i.)	*P*	OR (95% c.i.)	*P*
Age (years)	1.01 (0.99,1.03)	0.490	–	–
**Sex**				
Male	1 (reference)	–	–	–
Female	1.15 (0.54,2.44)	0.721	–	–
Number of metastases	0.87 (0.79,0.96)	0.005	0.92 (0.77,1.12)	0.369
**Number of affected segments**				
1	1 (reference)	–	1 (reference)	–
2	0.87 (0.21,3.61)	0.844	0.54 (0.09,3.21)	0.502
3	0.58 (0.13,2.70)	0.491	0.84 (0.18,4.02)	0.829
≥4	0.24 (0.08,0.77)	0.016	0.85 (0.17,4.36)	0.847
Number of chemotherapy cycles	0.92 (0.83,1.00)	0.068	–	–
**Procedure**				
Minor hepatectomy	1 (reference)	–	1 (reference)	–
Major hepatectomy	0.21 (0.09,0.46)	<0.001	0.54 (0.19,1.55)	0.254
**Approach**				
Open	1 (reference)	–	–	–
Laparoscopic	3.08 (1.30,7.31)	0.010	1.61 (0.57,4.54)	0.369
Previous portal embolization	0.09 (0.03,0.29)	<0.001	0.23 (0.06,0.93)	0.040
Two-stage surgery	0.28 (0.10,0.74)	0.011	0.99 (0.99,1.00)	0.131
Intraoperative blood loss (ml)	0.99 (0.99,1.00)	0.001	0.953 (0.21,4.29)	0.952

### Liver-first approach-specific textbook outcome

The patients who achieved an LFA-specific TO (69 patients, 46 per cent) were compared with those who did not (80 patients, 534 per cent) (*Table 1*). Significant differences were found regarding the number of metastases (two *versus* five respectively; *P* < 0.001), the size of the largest metastasis (23.5 *versus* 36 mm respectively; *P* = 0.001), the number of affected segments (for example if greater than four affected segments, 22 (32 per cent) *versus* 49 (61 per cent) respectively; *P* = 0.001), the number of chemotherapy cycles (5 *versus* 7 respectively; *P* = 0.007), and the type of procedure with LFA-specific TO being more frequent for patients undergoing minor hepatectomies (50 *versus* 39; *P* = 0.003). A total of 30 (57 per cent) patients treated laparoscopically achieved an LFA-specific TO, whereas a total of 39 (41 per cent) patients treated with an open approach achieved an LFA-specific TO (*P* = 0.044). Patients who underwent portal embolization had lower LFA-specific TO rates (15 (19 per cent) patients who did not achieve an LFA-specific TO *versus* 4 (6 per cent) patients who achieved an LFA-specific TO; *P* = 0.018). Intraoperative blood loss was greater for patients who did not achieve an LFA-specific TO compared with those who achieved an LFA-specific TO (350 *versus* 200 ml respectively; *P* = 0.018), surgical time was longer for patients who did not achieve an LFA-specific TO compared with those who achieved an LFA-specific TO (270 *versus* 210 min respectively; *P* < 0.001), and pedicle clamping time was longer for patients who did not achieve an LFA-specific TO compared with those who achieved an LFA-specific TO (40 *versus* 28 min respectively; *P* = 0.028).

Univariable analysis showed that number of metastases, size of the largest metastasis, number of affected segments, number of chemotherapy cycles, type of procedure, surgical approach, previous portal embolization, intraoperative blood loss, and pedicle clamping time were significant predictive factors for LFA-specific TO achievement (*Table 3*). In the multivariable analysis, only the number of metastases and intraoperative blood loss reached statistical significance (OR 0.82 (95 per cent c.i. 0.73 to 0.92) and OR 0.99 (95 per cent c.i. 0.99 to 1.00) respectively).

**Table 3:** Logistic regression analysis of characteristics associated with a liver-first approach-specific textbook outcome

Characteristic	Univariable analysis		Multivariable analysis	
	OR (95% c.i.)	*P*	OR (95% c.i.)	*P*
Age (years)	0.98 (0.96,1.03)	0.133	–	–
**Sex**				
Male	1 (reference)	–	–	–
Female	1.26 (0.64,2.49)	0.510	–	–
Number of metastases	0.82 (0.73,0.92)	0.001	0.86 (0.76,0.97)	0.027
Size of the largest metastasis (mm)	0.98 (0.97,0.99)	0.012	0.995 (0.98,1.01)	0.558
**Number of affected segments**				
1	1 (reference)	–	1 (reference)	–
2	0.63 (0.21,1.89)	0.413	1.63 (0.43,6.15)	0.468
3	0.31 (0.09,1.12)	0.075	0.50 (0.09,2.83)	0.436
≥4	0.18 (0.07,0.48)	0.001	0.89 (0.16,5.12)	0.897
Number of chemotherapy cycles	0.88 (0.79,0.98)	0.016	0.92 (0.8,1.05)	0.222
**Procedure**				
Minor hepatectomy	1 (reference)	–	1 (reference)	–
Major hepatectomy	0.36 (0.18,0.71)	0.004	0.75 (0.27,2.09)	0.579
**Approach**				
Open	1 (reference)	–	–	–
Laparoscopic	1.90 (0.97,3.76)	0.058	–	–
Previous portal embolization	0.25 (0.08,0.81)	0.020	0.53 (0.12,2.34)	0.406
Intraoperative blood loss (ml)	0.99 (0.99,1.00)	0.001	0.99 (0.99,1.00)	0.017
Pedicle clamping time (min)	0.98 (0.97,1.00)	0.040	0.99 (0.98,1.01)	0.782

**Fig. 1:**
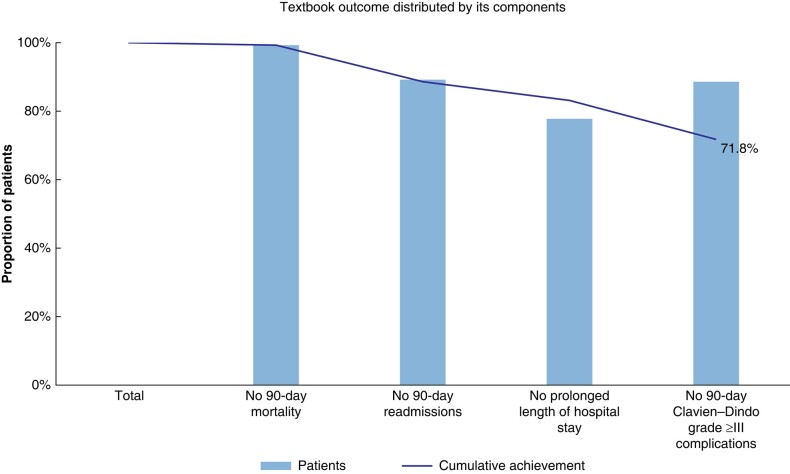
Distribution of a classic textbook outcome and its parameters

**Fig. 2:**
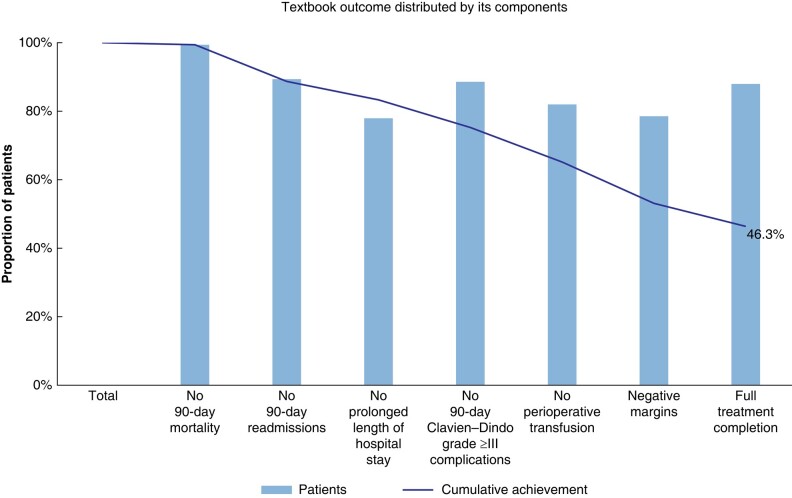
Distribution of a liver-first approach-specific textbook outcome and its parameters

Prolonged LOS (33 patients, 79 per cent) had the greatest negative impact on classic TO achievement (as shown in *Fig. 1* and *Table 4*), whereas prolonged LOS (33 patients, 41 per cent), positive margins (31 patients, 39 per cent), perioperative transfusion (27 patients, 34 per cent), and no full treatment completion (18 patients, 23 per cent) most frequently prevented LFA-specific TO achievement (as shown in *Fig. 2* and *Table 4*).

**Table 4:** Events triggering failure to achieve a textbook outcome

Characteristic	No classic textbook outcome (*n* = 42)	No liver-first approach-specific textbook outcome (*n* = 80)
Ninety-day Clavien–Dindo grade ≥III complications	17 (41)	17 (21)
Ninety-day mortality	1 (2)	1 (1)
Ninety-day readmissions	16 (38)	16 (20)
Length of hospital stay >9 days	33 (79)	33 (41)
Perioperative transfusion	–	27 (39)
Positive margins	–	31 (39)
No full treatment completion	–	18 (23)

Values are *n* (%).

## Discussion

Due to the absence of an international consensus definition of TO for liver surgery, several proposals have been published^3,5,8,10^. Some authors, for example de Graaff *et al*.^26^ and Görgec *et al*.^27^, have proposed to include the analysis of surgical resection margins.

The present study is, to the best of the authors’ knowledge, the first to examine TO as a quality measure with regard to the LFA for SCRLM. The classic TO rate observed in the present study is comparable to those of Görgec *et al*.^10^ (68 per cent) and de Graaff *et al*.^26^ (80 per cent), whereas the LFA-specific TO rate (46.3 per cent) is lower than those reported in the literature^6,10,19,27,28^. This could be explained by the different protocols used among centres, such as the Enhanced Recovery After Surgery (‘ERAS’) protocol, which takes into account LOS and readmission rate, both of which are TO parameters.

R0 resection continues to be the standard of care in liver surgery for SCRLM due to the implications regarding long-term outcomes^10^. R0 resection margins had the most negative effect on the achievement of a TO in liver surgery, together with prolonged LOS^10,18^.

The absence of studies measuring LFA-specific TOs and the heterogeneity of TO criteria (for example inclusion of other liver disease, such as cholangiocarcinoma) made it difficult to compare the results of the present study with those in the literature^6,10,19,27,28^. Comparing patients with colorectal liver metastases (CRLM) and HCC, Gorgec *et al*.^10^ showed that patients with CRLM achieved a TO significantly less frequently than patients with HCC.

Multicentre studies present a vast difference in the TO rates achieved among centres, ranging from 16.6 to 78.7 per cent^5,8,10,16,18^, without a correlation with procedure volume^10^. In another series of patients with CRC and SRCLM, a TO was achieved in 59 per cent of the patients and was associated with significantly improved overall and disease-free survival^3^.

The laparoscopic approach has been identified as an important factor for obtaining a TO^10,15,16^ and was the sole independent predictor associated with a TO for patients with HCC in Azoulay *et al*.^16^. The type of hepatectomy has also been related to TO achievement^5,16^. Tsilimigras *et al*.^18^ found that the highest TO rates were recorded for patients who underwent minor resections (73.0 per cent), whereas the lowest TO rates were recorded for patients undergoing major resections (34.0–43.0 per cent).

According to the multivariable regression analysis, the number of metastases and intraoperative blood loss were significantly associated with LFA-specific TO achievement. Both variables probably define patients with a high tumour burden who require complex hepatectomies and are typically treated using an LFA. In other series regarding TOs in liver surgery, age under 60 years, female sex, tumours smaller than 30 mm, and the absence of co-morbidities were associated with TO achievement^5,10^.

The present study has some limitations: liver resections were heterogeneous; and the sample size was limited. On the other hand, the follow-up interval was short and subsequently a survival/disease-free survival analysis has not been performed.

In conclusion, LFA-specific TO is a promising tool for measuring the quality of care in hospitals where this technique is performed; it may help to predict the clinical management of patients and, consequently, to limit costs.

## Collaborators

### RENACI Project Collaborative Study Group

Carlos Domingo-del-Pozo (Hospital Universitario Dr. Peset, Valencia, Spain); Gabriel García-Plaza (Hospital Universitario Insular, Las Palmas de Gran Canaria, Spain); Francisco J. González-Rodríguez (Hospital Clínico Universitario de Santiago, Santiago de Compostela, Spain); Eva M. Montalvá-Orón (Hospital Universitario y Politécnico La Fe, IIS La Fe, Ciberehd ISCIII, Valencia, Spain); Ángel Moya-Herraiz (Hospital Universitario de Castellón, Castellón, Spain); Sandra Paterna-López (Hospital Universitario Miguel Servet, Zaragoza, Spain); Miguel A. Suárez-Muñoz (Hospital Universitario Virgen de la Victoria, Málaga, Spain); Maialen Alkorta-Zuloaga (Hospital Universitario Donostia, San Sebastián, Spain); Gerardo Blanco-Fernández (Hospital Universitario de Badajoz, Badajoz, Spain); Enrique Dabán-Collado (Hospital Universitario San Cecilio, Granada, Spain); Miguel A. Gómez-Bravo (Hospital Universitario Virgen del Rocío, Sevilla, Spain); José I. Miota-de-Llamas (Hospital Universitario de Albacete, Albacete, Spain); Fernando Rotellar (Clínica Universidad de Navarra, Pamplona, Spain); Santiago Sánchez-Cabús (Hospital Universitario de la Santa Creu i Sant Pau, Barcelona, Spain); David Pacheco-Sánchez (Hospital Universitario Río Hortega, Valladolid, Spain); Juan C. Rodríguez-Sanjuán (Hospital Universitario Marqués de Valdecilla, Santander, Spain); María A. Varona-Bosque (Hospital Universitario Nuestra Señora de la Candelaria, Santa Cruz de Tenerife, Spain); Lucía Carrión-Álvarez (Hospital Universitario de Fuenlabrada, Madrid, Spain); Sofía de la Serna-Esteban (Hospital Clínico Universitario, Madrid, Spain); Cristina Dopazo-Taboada (Hospital Universitario Vall d’Hebron, Barcelona, Spain); Elena Martín-Pérez (Hospital Universitario La Princesa, Madrid, Spain); David Martínez-Cecilia (Hospital Universitario Virgen de la Salud, Toledo; Hospital Universitario La Princesa, Madrid, Spain); María J. Castro-Santiago (Hospital Universitario Puerta del Mar, Cádiz, Spain); Dimitri Dorcaratto (Hospital Clínico Universitario, Valencia, Spain); Marta L. Gutiérrez-Díaz (Hospital Quirón, Zaragoza, Spain); José M. Asencio-Pascual (Hospital Universitario Gregorio Marañón, Madrid, Spain); Fernando Burdío-Pinilla (Hospital Universitario del Mar, Barcelona, Spain); Roberto Carracedo-Iglesias (Hospital Universitario Álvaro Cunqueiro, Vigo, Spain); Alfredo Escartín-Arias (Hospital Universitario Arnau de Vilanova, Lleida, Spain); Benedetto Ielpo (Hospital Universitario de León, León; Hospital Universitario del Mar, Barcelona, Spain); Gonzalo Rodríguez-Laiz (Hospital General Universitario Dr. Balmis, Alicante, Spain); Andrés Valdivieso-López (Hospital Universitario de Cruces, Barakaldo, Spain); Emilio De-Vicente-López (Hospital Universitario HM Sanchinarro, Madrid, Spain); Vicente Alonso-Orduña (Hospital Universitario Miguel Servet, Zaragoza).

## Data Availability

The data sets used and/or analysed during the present study are available from the corresponding author on reasonable request.
